# 

**Published:** 1937

**Authors:** 


### The Bristol
### Medico-Chirurgical Journal

A JOURNAL OF THE MEDICAL SCIENCES FOR THE
WEST OF ENGLAND AND SOUTH WALES.

PUBLISHED UNDER THE AUSPICES OF
*THE BRISTOL MEDICO-CHIRURGICAL SOCIETY.*

EDITOR:
J. A. NIXON, C.M.G., M.D., F.R.C.P.

WITH WHOM ARE ASSOCIATED

E. W. HEY GROVES, D.Sc., M.S., F.R.C.S.
A. E. ILES, O.B.E., M.B., B.S., F.R.C.S.
A. RENDLE SHORT, M.D., B.S., B.Sc., F.R.C.S.
E. WATSON-WILLIAMS, M.C., Ch.M., F.R.C.S., Assistant Editor.

EDITORIAL SECRETARY:
A. L. FLEMMING, M.B., Ch.B.

"Scire est nescire, nisi id me
Scire alius sciret."

*VOL. LIV.*

BRISTOL: J. W. ARROWSMITH LTD.
London: J. W. Arrowsmith (London) Ltd.

### Contents of Vol. LIV

*Page*

The Evolution of a Casualty Clearing Station on the Western Front. By RICHARD C. CLARKE, O.B.E., M.B., F.R.C.P. .. .. 1

The Enlarged Prostate from the point of view of Practitioner and Surgeon. By C. FERRIER WALTERS, F.R.C.S. .. .. .. 21

The Therapeutic Value of Altitude. By BERNARD HUDSON, M.D., M.R.C.P. .. .. .. .. .. .. .. .. .. 35

The Rôle of the Central Nervous System in Disease: Recent Experimental Work in the U.S.S.R. By F. BODMAN, M.D. .. .. 41

The Carey Coombs Memorial Lecture on the Pathology and Surgical Treatment of Cardiac Ischæmia. By LAURENCE O'SHAUGHNESSY, F.R.C.S. .. .. .. .. .. .. .. .. .. 109

Some Reflections on Research in Medicine. By F. J. POYNTON, M.D., F.R.C.P. .. .. .. .. .. .. .. .. .. 127

Spinal Anæsthesia. By A. W. ADAMS, F.R.C.S. .. .. .. 143

How Bristol Royal Infirmary has Watched the World Change. By E. WATSON-WILLIAMS, M.C., F.R.C.S. .. .. .. .. 167

Nutrition and Tuberculosis. By Prof. J. A. NIXON, C.M.G., M.D., F.R.C.P. .. .. .. .. .. .. .. .. .. 191

The Burden Mental Research Trust: its Present and Future. By R. J. A. BERRY, M.D., F.R.S.E. .. .. .. .. .. 201

Sulphanilamide in Treatment. By E. WATSON-WILLIAMS, M.C., CH.M., F.R.C.S. .. .. .. .. .. .. .. .. .. 209

Prontosil in Obstetrics. By H. J. DREW-SMYTHE, M.S., F.R.C.S., F.C.O.G. .. .. .. .. .. .. .. .. .. 217

Ham Green Hospital Extensions. The Opening Address delivered by Prof. W. W. JAMESON, M.D., F.R.C.P., D.P.H., Barrister-at-Law 221

The Twenty-sixth Long Fox Memorial Lecture: The Debt of Western Medicine to the East. By V. B. GREEN-ARMYTAGE, M.D., F.R.C.P., F.C.O.G. .. .. .. .. .. .. .. 239

Child Guidance. By R. F. BARBOUR, M.B., CH.B., M.R.C.P., D.P.M. 261

A Lump in the Throat. By E. WATSON-WILLIAMS, M.C., CH.M., F.R.C.S. .. .. .. .. .. .. .. .. .. 279

The Bristol Royal Infirmary Bicentenary .. .. .. .. 287

REVIEWS OF BOOKS .. .. .. .. .. .. 64, 171, 227, 301

EDITORIAL NOTES .. .. .. .. .. .. 78, 180, 231, 313

OBITUARIES .. .. .. .. .. .. .. .. 82, 183

MEETINGS OF SOCIETIES .. .. .. .. .. 91, 184, 318

THE MEDICAL LIBRARY OF THE UNIVERSITY OF BRISTOL 92, 185, 234, 321

PUBLICATIONS RECEIVED .. .. .. .. .. 94, 187, 236, 323

LOCAL MEDICAL NOTES .. .. .. .. .. 96, 188, 237, 323

THE BRISTOL MEDICAL DRAMATIC CLUB .. .. .. .. .. 98

Mr. H. K. Pridie then showed a cinematograph film demonstrating the Hey Groves method of introduction of the modified Smith-Peterson pin in the treatment of intra-capsular fracture of the neck of the femur.

### The Medical Library of the University of Bristol,

WITH WHICH IS INCORPORATED

**The Library of the Bristol Medico-Chirurgical Society.**

*The following donations have been received since the publication of the list in January,* 1937.

March, 1937.

| | | |
|---|---|---|
| General Medical Council (1) .. .. .. | 3 | volumes. |
| Professor E. W. Hey Groves (2) .. .. | 6 | ,, |
| Dr. J. J. S. Lucas (3) .. .. .. .. | 5 | ,, |
| Dr. J. D. Powell (4) .. .. .. .. | 18 | ,, |
| Dr. Gustave Roussy (5) .. .. .. | 1 | volume. |
| University of Sheffield (6) .. .. .. | 1 | ,, |
| Dr. J. O. Symes (7) .. .. .. .. | 1 | ,, |
| Mitchell Clarke Fund (8) .. .. .. | 1 | ,, |
| Royal College of Physicians (9) .. .. | 1 | ,, |

Unbound periodicals have also been received from Professor E. W. Hey Groves, Professor C. Bruce Perry and Dr. J. Odery Symes.

### THE ONE HUNDRED AND FIFTY-EIGHTH LIST OF BOOKS.

The figures in round brackets refer to the figures after the names of the donors. The books to which no such figures have been attached have either been bought from the Library Fund or received through the *Journal.*

| | | |
|---|---|---|
| **Bayly M. Beddoe** .. | *Diet in relation to disease* .. .. .. .. | 1936 |
| **Billing, Archibald** .. | *First principles of medicine* .. 4th Ed. (4) | 1854 |
| **Black, Arthur D.** .. | *Operative dentistry* .. 7th Ed. Vol. 4 (8) | 1936 |
| **Bradbury, F. C. S.** .. | *Causal factors in Tuberculosis* .. .. .. | 1933 |
| *British Medical Association.* | *The British Medical Association's Proposals for a General Medical Service for the Nation* .. .. .. .. .. .. | 1930 |

**Burns, B. H. and Ellis, V. H.** *Recent Advances in Orthopædic Surgery* .. .. .. .. .. .. .. .. (2) 1937
**Chambers, Thomas King.** *The Indigestions* .. .. .. 2nd Ed. (4) 1867
**Chambers, Thomas King.** *The Renewal of Life* .. .. 2nd Ed. (4) 1863
**Chase, Robert Howland** *General Paresis, Clinical and Practical* (4) 1902
**Churchill, Fleetwood** *On the Theory and Practice of Midwifery* 6th Ed. (3) 1872
**Cohen S. Solis** (ed.) *A System of Physiologic Therapeutics* Vols. 1–4, 6 and 10 (3) 1901–1903
**Dixon, W. E.** .. .. *A Manual of Pharmacology* 8th Ed. revised by W. A. M. Smart 1936
*Engineers' Study Group on Economics. Interim Report on the Design of a Family Budget with Special Reference to Food* .. .. .. 2nd revised edition 1936
**Goodall, E. W.** .. *William Budd, M.D. Edin., F.R.S.* .. .. 1936
**Gordon, R. G., Harris, N. G., and Rees, J. R.** *An Introduction to Psychological Medicine* .. .. .. .. .. 1936
**Greene, Charles Lyman** *Medical Examination for Life Insurance* (4) 1902
**Hamilton, James** .. *Observations on the Utility and Administration of Preventive Medicines in Special Diseases* 4th Ed. (4) 1811
**Hickman, Henry Hill** *Centenary Exhibition—Souvenir* .. .. (2) 1930
**Inglis, K.** *Paget's Disease of the Nipple* .. .. .. 1936
**Jamieson, E. B.** .. *Illustrations of Regional Anatomy.* Sections VI. and VII .. .. .. .. .. .. .. .. .. 1936
**Jenkins, H. P.** .. .. *A Terminology of Operations of the University of Chicago Clinics* .. .. .. .. .. (2) 1935
**Kitchin, D. H.** .. .. *Legal Problems in Medical Practice* .. .. 1936
*Kodak X-ray Manual* .. .. .. .. .. .. .. .. .. .. .. .. .. (2) 1936
**Lambotte, Albin** .. *Livre Jubilaire* .. .. .. .. .. .. .. .. (2) 1936
*League of Nations.* .. *The Problem of Nutrition,* Vol. III .. .. 1936
**MacAlpine** .. .. .. *Cystoscopy and Urography* 2nd Ed. revised 1936
**Maw and Son Ltd.** .. *Catalogue of Surgical Instruments and Appliances* .. .. .. .. .. .. .. .. .. (4) 1913
**Medico, G. del** .. *Anatomia* .. .. .. .. .. .. .. .. .. .. (8) 1811
**Miles, A., and Withie, D.P.D.** *Operative Surgery* .. 2nd Ed. 1936
**Paget, George E.** .. *Some Lectures* .. .. .. .. .. .. .. .. (4) 1893
*Paris, Université de.* *Institut de Cancer* .. .. .. .. .. .. (5) 1936
**Pigg, T. Strangeways** *Clinical Pathology and Practical Morbid Histology* .. .. .. .. .. 2nd Ed. (4) 1901
**Plimmer, R. H. A.** .. *Analyses and Energy Values of Food* .. 1921
*Reports on Chronic Rheumatic Diseases.* Ed. by C. W. Buckley. No. 2 1936
**Rinehart, M. R.** .. *The Doctor* (fiction) .. .. .. .. .. .. .. 1936
**Roget, Peter Mark** .. *Animal and Vegetable Physiology* 2 vols. (4) 1834
**Roosen, R.**.. .. .. *A Theory of Cancer and Mnemotherapy* .. 1936
**Savage, George Henry** *The Increase of Insanity* .. .. .. (4) 1907
**Savill, T. D.** .. .. *A System of Clinical Medicine* 10th Ed. 1936
**Simpson, T. Levy** .. *Medical Diagnosis, some Clinical Aspects* .. 1937

| | | |
|---|---|---|
| *Southend General Hospital.* | *Contributions to Clinical Practice in Medicine and Surgery,* Ed. by T. Rowland Hill. Vol. I .. .. .. .. .. .. .. .. | 1936 |
| **Stevens, W. M.** .. .. | *The Prevention and Treatment of Disease* .. | 1936 |
| **Tchaperoff, I. C. C.** | *A Manual of Radiological Diagnosis* .. (2) | 1937 |
| **Thomas, Robert** .. | *The Modern Practice of Physic* 7th Ed. (3) | 1821 |
| **Thompson, K.** .. .. | *Elementary Pathology* .. .. .. .. .. .. | 1936 |
| **Thompson, T.** .. .. | *Annals of Influenza from* 1510 *to* 1837 (3) | 1852 |
| **Troup, W. Annandale** | *Therapeutic Uses of Infra-red Rays.* 3rd Ed. (7) | 1936 |
| **Turnbull, William** .. | *An Enquiry into the Origin and Antiquity of Lues Venerea* .. .. .. .. 7th Ed. (3) | 1805 |
| **Weber, F. P.** .. .. | *Some Thoughts of a Doctor* .. .. .. .. .. | 1935 |

TRANSACTIONS, REPORTS, JOURNALS, ETC.

| | |
|---|---|
| Brompton Hospital Reports. Vol. V. .. .. .. .. .. .. .. .. .. .. | 1936 |
| Dentists' Register, 1937 .. .. .. .. .. .. .. .. .. .. .. .. (1) | 1937 |
| Royal College of Physicians. List of fellows and members, 1937 (9) | 1937 |
| Medical and Dental Students Register, 1936 .. .. .. .. .. (1) | 1937 |
| Medical Register, 1937 .. .. .. .. .. .. .. .. .. .. .. .. (1) | 1937 |
| Sheffield, University of. Collected reprints of the School of Medicine (6) | 1934–1935 |
| Surgical Clinics of N. America. December, 1936 .. .. .. .. .. | 1936 |
| Transactions of the Association of American Physicians. Vol. LI. .. | 1936 |
| Transactions of the Ophthalmological Society of the United Kingdom. Vol. LVI... .. .. .. .. .. .. .. .. .. .. .. .. .. .. .. | 1936 |

---

### Publications Received

From EDWARD ARNOLD & CO. LTD.:

*Vade Mecum of Medical Treatment.* By W. GORDON SEARS, M.D., M.R.C.P. Price 10s. 6d.

From BAILLIÈRE, TINDALL & COX:

*Safe Childbirth: The Three Essentials.* By KATHLEEN O. VAUGHAN, M.B.Lond. Price 7s. 6d.

From JOHN BALE, SONS & DANIELSSON LTD.:

*The Main Points of Chemical Warfare from the Medical Aspect.* By K. SMALLCROSS DICKINSON, M.R.C.S., L.R.C.P., Ph.C. Price 1s.

*A Doctor at Work and Play.* By SIDNEY H. SNELL., M.D., B.S., D.P.H. Price 12s. 6d.

From CASSELL & CO. LTD.:

*Diseases of the Eye.* By EUGENE WOLFF, M.B., B.S., F.R.C.S.Eng. Price 15s.

*Sick Children: Diagnosis and Treatment.* By DONALD PATERSON, B.A., M.D., F.R.C.P. Price 12s. 6d.

From WILLIAM HEINEMANN LTD. :

*Diatherapy.* By ELKIN P. CUMBERBATCH, M.A., B.M., D.M.R.E., F.R.C.P. 3rd Edition. Price 21s.

*A Textbook of Medical Bacteriology.* By R. W. FAIRBROTHER, D.Sc., M.D., M.R.C.P. Price 15s.

From H. K. LEWIS & CO. LTD. :

*Diagnosis and Non-Operative Treatment of the Diseases of the Colon and Rectum.* By GOTTWALD SCHWARZ, M.D., JACQUES GOLDBERGER, M.D., and CHARLES CROCKER, M.D. Price 40s.

*Practical Orthoptics in the Treatment of Squint.* By KEITH LYLE, M.A., M.D., F.R.C.S., and SYLVIA JACKSON, S.R.N. Price 12s. 6d.

*Contributions to Clinical Practice in Medicine and Surgery, by the Staff of Southend General Hospital.* Edited by T. ROWLAND HILL, M.D., M.R.C.P. Price 12s. 6d.

*Medical Diagnosis : some Clinical Aspects.* By S. LEVY SIMPSON, M.A., M.D., M.R.C.P.

From E. & S. LIVINGSTONE.

*Diagnosis and Treatment of Venereal Diseases.* By DAVID LEES, D.S.O., M.A., M.B., D.P.H., F.R.C.S., F.R.C.P., F.R.S.E. 3rd Edition. Price 15s.

*Diseases of the Nose, Throat and Ear.* By I. SIMPSON HALL, M.B., Ch.B., F.R.C.P., F.R.C.S. Price 10s. 6d.

From LONDON VEGETARIAN SOCIETY :

*Diet in Relation to Disease : the Case for Vegetarianism.* By M. BEDDOW BAILY, M.R.C.S., L.R.C.P.

From OXFORD UNIVERSITY PRESS (HUMPHREY MILFORD) :

*Original Papers of Richard Bright on Renal Disease.* Edited by A. ARNOLD OSMAN, D.S.O., F.R.C.P. Price 21s.

*Hay Fever, with Special Reference to Treatment by Intranasal Ionization.* By CLIVE SHIELDS, B.M., B.Ch. Price 7s. 6d.

*A Social Problem Group.* Edited by C. P. BLACKER, M.C., M.A., M.D., F.R.C.P. Price 15s.

*The Facial Neuralgias.* By W. HARRIS, M.D., F.R.C.P. Price 7s. 6d.

*Charterhouse Rheumatism Clinic Original Papers,* Vol. I. Price 15s.

*High Blood Pressure.* By I. HARRIS, M.D. Price 10s. 6d.

From WILDING & SON, LTD. (Shrewsbury) :

*Obstetrical Technique.* By F. J. BROWNE, M.D., Ch.B., F.R.C.S., F.C.O.G. Price 2s.

From JOHN WRIGHT & SONS LTD. :

*Principles of Diagnosis, Prognosis and Treatment.* By R. HUTCHISON, M.D., LL.D., F.R.C.P. 2nd Edition. Price 3s. 6d.

*British Journal of Surgery.* (January, 1937.) Price 12s. 6d.

*Elements of Orthopædic Surgery.* By N. ROSS SMITH, M.B., Ch.M., F.R.C.S. Price 10s. 6d.

*Latent Syphilis and the Autonomic Nervous System.* By GRIFFITH EVANS, M.A., D.M. F.R.C.S., D.O.M.S. Price 7s. 6d.

### Local Medical Notes

EXAMINATION RESULTS.

**University of Bristol.**—Students of the University have recently passed the following examinations:—

M.B., Ch.B.—*Second Examination* (*Section II.*) *Pass :* R. G. Boyd (with Distinction in Anatomy), N. F. W. Brueton (with Distinction in Anatomy), T. J. Butler (with Distinction in Anatomy), G. T. Cribb, Winifred M. Curgenven-Robinson, Clara J. Fraser, G. H. Harvey, Gwendolen M. Higgins, Mary L. Hunt, P. Jacobs, Megan E. Jones, J. K. Lewis, H. R. Newman, J. W. J. Newton, Marjorie Organ, R. D. R. Shanks, Patricia M. Simpson, J. J. de S. Snijman, D. A. Squire, Barbara F. Thomas (with Distinction in Anatomy), Joan Threadgold, G. H. Tovey, J. B. Tucker, D. N. Walder, L. Willoughby.

B.D.S.—*Second Examination. Pass :* G. H. Bell, L. B. Howell.

L.D.S.—*Second Examination. Pass :* L. H. Barker-Tufft, K. A. Gordon-Ralph, G. H. Hudson, D. G. Davies, J. R. Herbert, P. Le Masurier, C. R. Sage.

**Conjoint Board.** — M.R.C.S., L.R.C.P. — *Pass : In Midwifery :* C. R. G. Howard, M. E. M. Herford, F. J. W. Hooper. *In Surgery and Midwifery :* K. C. P. Smith. *In Pathology :* J. R. G. Damrel, A. M. Spencer. *In Surgery* · F. D. Mowat.

L.D.S., R.C.S.—*First Professional Examination. Pass :* (*Dental Mechanics and Dental Metallurgy ; and Anatomy and Physiology*) *:* S. G. Sheffield. (*Dental Mechanics and Dental Metallurgy*) *:* J. F. Williams.

---

The twenty-sixth Long Fox Memorial Lecture will be delivered on Tuesday, October 19th, 1937, at 8.30 p.m. by Professor V. B. Green-Armytage, M.D., F.R.C.P., F.G.O.C.

The Carey Coombs Memorial Lecture was delivered on Wednesday, May 12th, at 8.30 p.m. in the Physiology Theatre of the University by Mr. Laurence O'Shaughnessy, F.R.C.S., on " The Surgical Treatment of Cardiac Ischæmia."

**Colston Research Society.**—The Thirty-third Annual Dinner of the Colston Research Society will be held in the Great Hall of the University of Bristol, on Friday, 28th May next, under the Presidency of The Right Hon. The Viscount Bledisloe, P.C., G.C.M.G., K.B.E. The guest of the evening will be Professor R. G. Stapledon, C.B.E., M.A., Professor of Agricultural Botany, University College of Wales, whose invaluable work in the field of plant research and pasture improvement is recognized among agriculturists throughout the world as being without parallel. For further particulars apply to the Hon. Secretary of the Colston Research Society, 12 Small Street, Bristol 1.

---

APPOINTMENT.

**Bristol Eye Hospital.**—Mr. Anthony Gurdon Palin, M.A., M.B., B.Ch.(Oxon), M.R.C.S.(Eng.), L.R.C.P. (Lond.), F.R.C.S. (Edinburgh), Assistant Surgeon.

---

**Assistant Editor, change of address.**—After April 30th all books reviewed, reviews, and other communications for the Assistant Editor should be addressed to Mr. E. Watson-Williams, at **65 Pembroke Road,** Clifton, Bristol.

### Bristol Medico-Chirurgical Society.

### President.

R. C. CLARKE, *O.B.E.*, M.B., Ch.B. Brist.

### President-Elect.

C. A. MOORE, M.B., M.S. (Lond.), F.R.C.S.

### Committee.

*Ex-Officio*

- J. A. NIXON, *C.M.G.*, B.A., M.D. Cantab., F.R.C.P. (Editor of *Journal*).

- (Vacant), (Hon. Librarian).

*Elected.*

H. ELWIN HARRIS, B.A., M.B. Cantab., F.R.C.S. (Ex-President).
DUNCAN WOOD, F.R.C.S.
A. L. FLEMMING, M.B., Ch.B. Brist. (Ex-President).
CLIFFORD A. MOORE, M.B., M.S. Lond.
H. L. FULLER.
P. S. MARTIN, *M.C.*
W. V. WOOD, *M.C.*, B.A. Cantab.
A. FELLS, M.B., C.M. Edin.
R. C. CLARKE, *O.B.E.*, M.B., Ch.B. Brist.
A. L. TAYLOR, M.D. Lond.

### Honorary Secretary.

R. V. COOKE, Ch.M.

### Honorary Treasurer.

A. E. ILES, *O.B.E.*, M.B., B.S. Lond., F.R.C.S.

### University Library Sub-Committee.

A. R. SHORT, B.Sc., M.D. Lond., F.R.C.S.
J. O. SYMES, M.D. Lond.

Medical Practitioners wishing to join the Society are requested to communicate with the Hon. Secretary, Mr. R. V. COOKE, Failand Lodge, Guthrie Road, Clifton. The Annual Subscription is £1 1s., payable to Hon. Treasurer.

*Extracts from Journal By-laws.*

4.—The *Journal* shall be delivered free to Subscribers and also to Members of the Society whose subscriptions are not in arrear.

6.—The Annual Subscription to the *Journal* is 10/6, payable to Hon. Treasurer.

Communications intended for insertion in the *Journal* should be sent to the Editor, Dr. J. A. NIXON, 7 Lansdown Place, Clifton, Bristol.

Books for Review and Exchange Journals should be sent to the Assistant-Editor, *Bristol Medico-Chirurgical Journal*, Medical Library, The University, Bristol. Reviews should be returned with the books to the Assistant Editor at 65 Pembroke Road, Clifton, Bristol 8.

Communications referring to the delivery of the *Journal* should be sent to the Editorial Secretary, A. L. Flemming, 48 Pembroke Road, Clifton, Bristol.

Cases for Binding Vols. I—LIII are now ready, and may be obtained price 1/9 each (postage extra), upon application to J. W. ARROWSMITH LTD., Quay Street, Bristol; or the *Journal* will be bound, including Case, for 7/- per volume. The inclusive index for Volumes I—L may be obtained, price 3/- (postage extra).

VOL. LIV. No. 203.

### Bristol Medico-Chirurgical Society.

### PAST PRESIDENTS.

1874—75. FREDERICK BRITTAN, M.D., B.S. Dub.
1875—76. ROBERT WILLIAM COE.
1876—77. SAMUEL MARTYN, M.D. Ed.
1877—78. AUGUSTIN PRICHARD, M.D. Berlin.
1878—79. HENRY EDWARD FRIPP, M.D. Würzburg.
1879—80. WILLIAM MICHELL CLARKE.
1880—81. JOSEPH GRIFFITHS SWAYNE, M.D. Lond.
1881—82. EDWARD LONG FOX, M.D. Oxon.
1882—83. JAMES GEORGE DAVEY, M.D. St. And.
1883—84. WILLIAM JOHNSTONE FYFFE, M.D., B.A. Dub.
1884—85. GEORGE FORSTER BURDER, M.D. Aberd.
1885—86. WILLIAM HENRY SPENCER, M.D., M.A. Cantab.
1886—87. FRANCIS POOLE LANSDOWN.
1887—88. LEMUEL MATTHEWS GRIFFITHS.
1888—89. ROBERT SHINGLETON SMITH, M.D., B.Sc. Lond.
1889—90. NELSON CONGREVE DOBSON, Ch.M. Brist.
1890—91. SAMUEL HENRY SWAYNE.
1891—92. FRANCIS RICHARDSON CROSS, M.B. Lond., LL.D. Brist.
1892—93. EDWARD MARKHAM SKERRITT, M.D., B.S., B.A. Lond.
1893—94. JAMES GREIG SMITH, M.B., C.M., M.A. Aberd., F.R.S.E.
1894—95. ALFRED JAMES HARRISON, M.B. Lond.
1895—96. ARTHUR WILLIAM PRICHARD.
1896—97. ALFRED EDWARD AUST LAWRENCE, M.D., C.M. Aberd.
1897—98. JOHN EDWARD SHAW, M.B., C.M. Ed.
1898—99. ROBERT ROXBURGH, M.B. Ed.
1899—00. WILLIAM HENRY HARSANT.
1900—01. DAVID SAMUEL DAVIES, M.D. Lond.
1901—02. BARCLAY JOSIAH BARON, M.B., C.M. Ed.
1902—03. GEORGE MUNRO SMITH, M.D. Brist.
1903—04. JAMES PAUL BUSH, C.M.G., C.B.E., Ch.M. Brist.
1904—05. REGINALD EAGER, M.D. Lond.
1905—06. JOHN DACRE.
1906—07. JAMES TAYLOR.
1907—08. HENRY WALDO, M.D., C.M. Aberd.
1908—09. JOHN MICHELL CLARKE, M.D., M.A. Cantab.
1909—10. JAMES SWAIN, C.B., C.B.E., M.D., M.S. Lond.
1910—11. BERTRAM MITFORD HERON ROGERS, M.D., B.Ch., B.A. Oxon.
1911—12. CHARLES ALEXANDER MORTON.
1912—13. WALTER CARLESS SWAYNE, M.D., B.S. Lond. and Brist.
1913—14. PATRICK WATSON-WILLIAMS, M.D. Lond., M.D. (Hon. Causa) Brist.
1914—15. WILLIAM HARRY CHRISTOPHER NEWNHAM, M.A., M.B. Cantab.
1915—16. GEORGE PARKER, M.A., M.D. Cantab., LL.D. Brist.
1916—17. FRANCIS HENRY EDGEWORTH, M.A., M.D. Cantab., D.Sc. Lond.
1917—18. FREDERICK LACE.
1918—19. ROBERT GUTHRIE POOLE LANSDOWN, M.D., B.S. Durh.
1919—20. I. WALKER HALL, M.D., Ch.B. Vict.
1920—21. LEOPOLD ERNEST VALENTINE EVERY-CLAYTON, M.D. Lond.
1921—22. CYRIL HUTCHINSON WALKER, B.A., M.B. Cantab.
1922—23. JOHN ALEXANDER NIXON, C.M.G., B.A., M.D. Cantab.
1923—24. JOHN LACY FIRTH, M.D., M.S. Lond.
1924—25. JOHN ODERY SYMES, M.D. Lond.
1925—26. THOMAS CARWARDINE, M.S. Lond.
1926—27. ARTHUR LAUNCELOT FLEMMING, M.B., Ch.B. Brist.
1927—28. WALTER KENNETH WILLS, M.A., M.B., B.Ch. Cantab.
1928—29. HENRY LAWRENCE ORMEROD, M.D., B.Ch. R.U.I.
1929—30. CHARLES FERRIER WALTERS.
1930—31. DAVID CHARLES RAYNER, Ch.M. Brist.
1931—32. JOHN ROGER CHARLES, M.A., M.D. Cantab.
1932—33. ERNEST WILLIAM HEY GROVES, M.D., M.S., D.Sc., B.Sc. Lond.
1933—34. HERBERT ELWIN HARRIS, B.A., M.B. Cantab.
1934—35. A. RENDLE SHORT, B.Sc., M.D. Lond.
1935—36. ARTHUR ERNEST ILES, O.B.E., M.B., B.S. Lond.

1937.

### LIST OF SUBSCRIBERS

TO THE

### Bristol Medico-Chirurgical Journal.

### HONORARY MEMBERS OF THE SOCIETY.

| | |
|---|---|
| SWAIN, JAMES, *C.B.*, *C.B.E.*, M.D., M.S. Lond. .. .. .. .. | Wyndham House, Easton-in-Gordano. |
| WATSON-WILLIAMS, P., M.D. Lond. | 2 Rodney Place, Clifton, Bristol 8. |

### SUBSCRIBERS.

*Those marked * are Members of the Society.*

| | |
|---|---|
| *ACKLAND, W. R., M.D.S. .. .. .. .. .. .. | 5 Rodney Place, Clifton, Bristol 8. |
| *ADAMS, A. W., M.S. Lond. .. .. .. .. | Rodney House, Clifton, Bristol 8. |
| ADAMS, J. B .. .. .. .. .. .. .. .. .. | Kent Lodge, Eastbourne. |
| *ADAMS, S.B., Ph.D., M.B., Ch.B. Brist. .. .. | 19 Charlotte Street, Bristol 1. |
| *ALDWINCKLE, HELEN M., M.B., Ch.B. Brist. .. | 1 Manor Road, Fishponds, Bristol. |
| *ALEXANDER, A. J. P., M.D. Belf. .. .. .. | Winsley Sanatorium, near Bath. |
| *ALEXANDER, D. A., M.B., Ch.B. Vict. .. .. | 112 Pembroke Road, Clifton, Bristol 8. |
| ALEXANDER, G. L., B.A., M.B., B.Ch. Cantab. | West African Medical Service, Laura, via Accra, Gold Coast. |
| *ALFORD, A. F., M.B., Ch.B. Brist. .. .. .. | Central Hotel, Bristol 1. |
| ALFORD, H. J., M.D. Lond. .. .. .. .. | 4 Park Terrace, Taunton. |
| *ARMSTRONG, JEAN, M.B., Ch.B. Brist. .. .. | Succoth, Duckmoor Road, Ashton Gate, Bristol 3. |
| ASHTON, L. P., M.B., Ch.B. Brist. .. .. .. | Kapsowar, P.O. Eldoret, Kenya, East Africa. |
| *AUBREY, T., M.B. Lond. .. .. .. .. .. | Bitton, near Bristol. |
| *BAIN, W. .. .. .. .. .. .. .. .. .. .. | 1 Greville Road, Southville, Bristol 3. |
| *BAKER, LILY A., M.D., B.Ch., B.A.O., R.U.I. | Clifton Down House, Beaufort Buildings, Clifton, Bristol 8. |
| *BARBER, M. C., M.B., Ch.B. Brist. .. .. .. | Ingledene, High Street, Staple Hill, Bristol. |
| *BARBOUR, R. F., M.A. (Cantab.), M.B., Ch.B. | 16 Mortimer Road, Clifton, Bristol. |
| *BARKER, G. H., M.D., B.Sc. Lond. .. .. .. | 124 Redland Road, Bristol 6. |
| *BARTHOLOMEW, E. U. .. .. .. .. .. .. .. | 12 Lansdown Place, Clifton, Bristol 8. |
| *BATES, R. M. .. .. .. .. .. .. .. .. .. | Purdown House, Stapleton, Bristol 5. |
| BEATSON, D. H., M.B., Ch.B. Brist. .. .. | 8 Richmond Park Road, Clifton, Bristol 8. |
| *BERGIN, F. G. .. .. .. .. .. .. .. .. .. | 31 Oakfield Road, Clifton, Bristol 8. |
| *BERRY, R. J. A., Prof., M.D., C.M. Edin. .. | Director of Medical Services, Stoke Park Colony, Bristol 5. |
| *BIRRELL, J. A., M.D., B.S. Lond. .. .. .. | 36 Kellaway Avenue, Bristol 6. |
| *BLACHFORD, J. V., *C.B.E.*, M.D. Durh. .. .. | Milverton House, Long Ashton, near Bristol. |
| BLACKETT, J. F., M.D., B.S. Lond. .. .. .. | Hamsterley, Bloomfield Park, Bath. |
| BLETCHLY, G. PLAYNE, M.B. Lond. .. .. | 35 Stratford Road, Summerton, Oxford. |
| *BODMAN, A. P., M.B., Ch.B. .. .. .. .. .. | 11 Hurle Crescent, Clifton, Bristol 8. |
| *BODMAN, C. O., M.D. Durh. .. .. .. .. .. | 17 Upper Belgrave Road, Clifton, Bristol 8. |
| *BODMAN, F. H., M.D., Ch.B. Brist. .. .. .. | 2 Redland Court Road, Bristol 6. |
| BOLT, R. F. .. .. .. .. .. .. .. .. .. .. | 70 Great Russell Street, London, W.C.1. |
| *BOULTON, B. J., M.B., Ch.B. Brist. .. .. | Ham Green Hospital, Pill, near Bristol. |
| *BOWN, NAOMI J., M.B., Ch.B. Brist. .. .. | Demeter, Wraxall, Somerset. |
| *BRADBEER, E. G., M.B., Ch.B. Brist. .. .. | 13 Percival Road, Bristol 8. |
| *BRADLEY, W. H., B.A., D.M. Oxon. .. .. | 11–12 Wimpole Street, London, W.1. |
| BRASHER, C. W. J. .. .. .. .. .. .. .. .. | 62 Queen Anne St., Cavendish Square, London, W.1. |
| *BRINCKMAN, WINIFRED P., M.B., Ch.B. Brist. | 4 Oakland Road, Redland, Bristol 6. |
| *BRITTON, R. B., M.B., B.S. Lond. .. .. .. | 33a Whiteladies Road, Clifton Bristol 8. |
| *BROCKLEHURST, R. J., M.A., D.M. Oxon. .. | The University, Bristol 8. |

| | |
|---|---|
| BROWN, J. E., M.B., Ch.B. Brist. | 137 Belmont Circular Road, Port of Spain, Trinidad, B.W.I. |
| *BUCKMASTER, G. A., M.A., M.D. Oxon. | 6 Victoria Square, Clifton, Bristol 8. |
| *BURGES, R. | Druid's Mead, Stoke Bishop, Bristol 9. |
| *BURGESS, N. F. C., M.D. Cantab. | Hereford House, Clifton, Bristol 8. |
| *BURNETT, R. H., M.B., B.S. Durh. | 528 Bath Road, Brislington, Bristol 4 |
| *BUSH, G. B., M.B., Ch.B. Brist. | 132 Queen's Road, Clifton, Bristol 8. |
| *CAIRNS, F. J. | Devon House, Whitehall Road, Bristol 5. |
| *CAPPER, W. M., M.B., B.S. Lond. | Bristol Royal Infirmary, Bristol. |
| *CAREY, R. S., *O.B.E.*, B.A. Cantab. | 502 Bath Road, Brislington, Bristol 4. |
| *CARLETON, H. H., M.A., M.D., B.Ch. Oxon. | 1 Lansdown Road, Clifton, Bristol 8. |
| *CASSON, E., M.D., Ch.B. Brist. | Dorset House, Clifton Down, Bristol 8. |
| *CATES, H. J., M.D. Lond. | Northwoods House, Winterbourne, Bristol. |
| *CHAMBERS, E. R. | 9 Clifton Park, Clifton, Bristol 8. |
| *CHARLES, J. R., M.A., M.D. Cantab. | 5 Rodney Place, Clifton, Bristol 8. |
| *CHITTY, H., M.S. Lond. | 46 Pembroke Road, Clifton, Bristol 8. |
| *CHRISTOFFERSON, P. E., M.B., Ch.B. Brist. | 4 Victoria Square, Clifton, Bristol 8. |
| *CLAREMONT, L. E., M.D.S. Brist. | 5 Rodney Place, Clifton, Bristol 8. |
| *CLARKE, R. C., *O.B.E.*, M.B., Ch.B. Brist. | 29 Victoria Square, Clifton, Bristol 8. |
| *CLUTTERBUCK, E. R., M.B., Ch.B. Brist. | 63 Walliscote Road, Weston-super-Mare. |
| *COCHRANE, J. H. | Royal Infirmary, Bristol. |
| *COLLINGWOOD, F. C., M.B., Ch.B. Brist. | Southmead Hospital. |
| CONNELLAN, P. S. | Marmion, Shortheath Road, Farnham, Surrey. |
| *COOKE, ELIZABETH M., M.D. Lond. | Failand Lodge, Guthrie Road, Clifton. |
| *COOKE, R. V., Ch.M. Brist. | Failand Lodge, Guthrie Road, Clifton. |
| *COOKSON, R. G. | 5 Worcester Road, Clifton, Bristol 8. |
| *COOMBS, C. F., B.Ch. Cantab. | General Hospital, Bristol. |
| *COOMBS, N. C. | 14 Southfield Road, Westbury-on-Trym Bristol. |
| *COOPER, W. R. | 13 Westfield Park, Redland, Bristol 6. |
| *CORFIELD, C., M.D. Durh. | 5 Kensington Place, Clifton, Bristol 8. |
| *CORNER, BERYL, M.D., B.S. Lond. | 3 Elton House, Rodney Place, Bristol 8. |
| *COSSHAM, W. L., M.B., Ch.B. Brist. | 3 Claremont Road, Bishopston, Bristol 7. |
| *COURTNEY, R. M., M.B., Ch.B. Glasg. | 487 Gloucester Road, Bristol 7. |
| *COXON, BEATRICE | 16 Richmond Hill, Clifton, Bristol 8. |
| *CRAIG, ANNE A., M.B., B.S. Lond. | 18 Vyvyan Terrace, Clifton, Bristol 8. |
| *CROOK, B. A., M.B., Ch.B. Brist. | Timsbury. |
| *CROSSMAN, F. W., M.B. Durh. | White's Hill, Hambrook, near Bristol. |
| *DATTA, S. S., M.D. Brist. | Snowdon House, Fishponds, Bristol. |
| *DAVIE, T. B., M.D. Liverpool | Canynge Hall, Clifton, 8. |
| *DAVIES, E. D. D. | Standish House, Stonehouse, Glos. |
| *DAWSON, W. R., *O.B.E.*, M.D. Dub. | 18 Brock Street, Bath. |
| *DELANEY, N., M.B., Ch.B. Manch. | The Meade, Bishopsworth, Somerset. |
| *DELICATI, J. L. | Royal Mineral Water Hospital, Bath. |
| DEVINE, H., *O.B.E.*, M.D. Lond. | Holloway Sanatorium, Virginia Water. |
| *DICK, A., M.B., Ch.B. Glasg. | Aldersyde, Birstall, Leeds. |
| *DICKSON, W. A., M.D. St. And. | Standish House, Stonehouse, Glos. |
| *DISNEY, M., M.D. Lond. | Bristol Royal Infirmary. |
| *DIX, C. | 11 Victoria Square, Clifton, Bristol 8. |
| *DIXON, HELEN M., M.B., Ch.B. Brist. | 10 Whatley Road, Clifton, Bristol 8. |
| *DIXON, T. B. | 11 Pembroke Road, Clifton, Bristol 8. |
| *DREW, J. H., M.D. Lond. | Penlee, Uphill Road, Weston-super-Mare. |
| *DUERDEN, J. R., M.B., Ch.B. Brist. | 149 Bell Hill Road, St. George, Bristol 5. |
| *DUNLEVY, A. A., M.B., B.Ch. N.U.I. | 23 Pembroke Road, Clifton, Bristol. |
| *DUTTON, HILDA R. | 405 Stapleton Road, Bristol 5. |
| *DYER, W. P. | West View, Westbury-on-Trym, Bristol. |
| *EGLINTON, J. H. C. | Street, Somerset. |
| ELLIOTTS & AUSTRALIAN DRUG LTD. | 29 O'Connell Street, Sydney, N.S.W., Australia. |
| ELLIOTT, F. P., M.B., C.M. Aberd. | 113 Grove Road, Walthamstow London, E. 17. |

| | |
|---|---|
| *ELLIOTT, W. H. A., M.B., B.S. Lond. | 116 Pembroke Road, Clifton, Bristol 8. |
| *ELLIS, W. T. | Tregenna House, Shirehampton. |
| *EYRE-BROOK, A. L., M.B., M.S. Lond. | The Cottage, Portishead. |
| *EVANS, G. M., M.B., Ch.B. Brist. | 117 Ashley Road, Bristol. |
| *FALLON, Col. J. | 35 Henleaze Gardens, Henleaze, Bristol. |
| *FARAKER, E. C. | 85 Coldharbour Road, Westbury Park, Bristol 6. |
| *FARQUHARSON, J. L. | Breda, Weston-super-Mare. |
| *FAULL, J. L., M.R.C.S., L.R.C.P., D.P.M. | 61 Cotham Brow, Bristol. |
| *FAWN, G. F. | 6 Oakfield Road, Clifton, Bristol 8. |
| *FELLS, A., M.B., C.M. Edin. | 6 Elton Road, Clifton, Bristol 8. |
| *FELLS, G. R., M.B., Ch.B. Brist. | 19 Southmead Road, Bristol. |
| *FELLS, R. R., M.B., B.S. Lond. | 17 Mortimer Road, Clifton, Bristol 8. |
| *FENELEY, G. L., M.B., Ch.B. Brist. | Englewood, Falcondale Road, Westbury-on-Trym. |
| *FINZEL, H. F. M., M.D. Brist. | Nare House, Birchwood Road, St. Anne's Park, Bristol 4. |
| *FIRTH, J. L., M.D., M.S. Lond. | 8 Victoria Square, Clifton, Bristol 8. |
| *FISHER, A. C., M.B., Ch.B. Brist. | Roan Antelope Mine, N. Rhodesia. |
| *FITZGIBBON, G. M., M.D. Lond. | 52 Pembroke Road, Clifton, Bristol 8. |
| *FITZGIBBON, Mrs. L. P., M.B., B.S. Lond. | 52 Pembroke Road, Clifton, Bristol 8. |
| *FLEMMING, A. A. G. | Manver's House, Bradford-on-Avon. |
| *FLEMMING, A. L., M.B., Ch.B. Brist. | 48 Pembroke Road, Clifton, Bristol 8. |
| *FORRESTER-BROWN, MAUD, M.D., M.S. Lond. | 22 Combe Park, Bath. |
| *FOSS, G. L., M.B., B.Ch. Cantab. | Cloud's Hill House, St. George, Bristol 5. |
| *FOX, F. E., B.A. Cantab. | Brislington House, Brislington, Bristol 4. |
| *FRASER, A. D., M.A., M.B., Ch.B. Glasg. | 4 Alexandra Road, Clifton, Bristol 8. |
| *FRASER, D., M.B., Ch.B. Edin. | Westgate, Dursley, Glos. |
| *FULLER, H. L. | 9 Gay Street, Bath. |
| *FURNIVAL, Col. C. H. | "Yatton," Wellington Hill, Horfield, Bristol 7. |
| *GARDEN, R. R., M.A., M.B., B.Ch. Aberd. | 9 Harley Place, Clifton, Bristol 8. |
| GARLAND, E. B., M.B., C.M. Edin. | 114a Hampton Road, Redland, Bristol 6. |
| *GEE, C. A. H., M.B. Ch.B. Brist. | Wansdyke, Portbury, near Bristol. |
| *GERRISH, NORMAN, M.B., Ch.B. Brist. | 330 Church Road, St. George, Bristol. |
| GIBBS, J. R. | 48 Codrington Road, Bishopston, Bristol 7. |
| *GLENNY, E. T., M.B., B.S. Lond. | 102 Pembroke Road, Clifton, Bristol 8. |
| *GORDON, R. G., M.D., B.Sc. Edin. | 2 Catharine Place, Bath. |
| *GORHAM, A. P., M.B., Ch.B. Brist. | 53 Westbury Road, Westbury-on-Trym. |
| *GORNALL, W. A., M.D. Brist. | Orchard House, Nailsea, Somerset. |
| *GRAY, J. D. A., M.B., Ch.B., B.Sc. Edin. | 212 Redland Road, Bristol 6. |
| *GREEN, S. B., M.B. Lond. | The Black Wicket, Westbury-on-Trym. |
| *GREENLAW, MADGE E., M.B., Ch.B. Brist. | 194 Redland Road, Bristol 6. |
| GRIFFITHS, CORNELIUS A. | 35 Newport Road, Cardiff. |
| *GROVES, E. W. HEY, M.D., M.S., D.Sc. Lond. | 25 Victoria Square, Clifton, Bristol 8. |
| *GUIRDHAM, A., M.A., B.M., B.Ch. Oxon. | Bailbrooke House, Bath. |
| *HALL, E. GEORGE, M.B. Lond. | 42 Apsley Road, Clifton, Bristol 8. |
| *HAM, C. I. M.B., Ch.B. Brist. | Frenchay Park Sanatorium, Frenchay, near Bristol. |
| *HARRIS, H. ELWIN, M.A., M.B. Cantab. | The Mount, Halse, Taunton. |
| *HARRIS, H. E., M.A., M.B., B.Ch. Cantab. | 13 Lansdown Place, Clifton, Bristol 8. |
| HARRISON, C. C. | 44 High Street, Keynsham. |
| *HARTLEY, G., M.B., B.S. Lond. | 2 Harley Place, Clifton, Bristol 8. |
| *HAWKINS, E. J. | 27 Westbury Road, Westbury-on-Tryn. |
| *HEALES, J. N. | Holmcroft, Street, Somerset. |
| *HECTOR, F., M.D. Lond. | 1 Victoria Square, Clifton, Bristol 8. |
| *HEMPHILL, R., M.B., B.Ch. Dub. | Fishponds Mental Hospital, Bristol. |
| *HENDERSON, JAMES, M.B., Ch.B. Aberd. | The City Sanatorium, Yardley Road, Birmingham. |
| *HERAPATH, C. E. K., *M.C.*, M.D. Lond. | Ormlie, Clifton, Bristol 8. |
| *HERON, GORDON, M.B., Ch.B. Brist. | 34 Gloucester Road, Bristol 7. |
| *HERON, DORIS, M.B., Ch.B. Brist. | 34 Gloucester Road, Bristol 7. |
| *HILL, WINIFRED, M.B., Ch.B. Brist. | Ilford Maternity Home, Eastern Avenue, Ilford, Essex. |

| | |
|---|---|
| *HOOKER, J. A., M.B., Ch.B. Brist. | 16 St. Edyth's Road, Sea Mills 9. |
| *HOSKEN, J. G. F. | Uley, Glos. |
| HOSPITAL, BRISTOL GENERAL (*Secretary*, T. W. GREGG). | |
| *HOUGHTON, LYDIA M., M.B., B.S. Lond. | 74 Church Road, Redfield, Bristol 5. |
| *HOWELL, H. | Kincraig, Beach Road, Weston-super-Mare. |
| *HUGHES, F. W. TERRELL, M.B., Ch.B. Brist. | Chew Magna, Somerset. |
| *HUGHES, MARGUERITE G., M.B., Ch.B. Brist. | Tower House, Whiteladies Road, Bristol. |
| *HUNTER, F. S. | c/o Messrs. J. Wright & Sons Ltd., Publishers, Bristol 1. |
| *HUTCHINSON, BRENDA | 23 The Circus, Bath. |
| *HUTCHINSON, C. A. | 23 The Circus, Bath. |
| *HYATT, ANNIE W., M.B., B.S. Lond. | Merrynead, Shepton Mallet, Som. |
| *ILES, A. E., *O.B.E.*, M.B., B.S. Lond. | 17 Victoria Square, Clifton, Bristol 8. |
| INFIRMARY, BRISTOL ROYAL (*Secretary*, E. C. SMITH). | |
| INGLE, C. DURBAN | Somerton, Somerset. |
| *JACKMAN, W. A., M.B., Ch.B. Brist. | 15 Mortimer Road, Clifton, Bristol 8. |
| *JAMES, APRIL D., M.B., Ch.B. Brist. | |
| *JAMES, J. A, M.D. Lond. | 5 Rodney Place, Clifton, Bristol 8. |
| *JAMES, J. A. Senr. | 43 Nevil Road, Bishopston, Bristol. |
| *JENKINS, F. G., M.B., Ch.B. Brist. | 51 Redcliff Hill, Bristol 1. |
| *JENKINS, R. D. | The Copper Beeches, Almondsbury, near Bristol. |
| *JOHNSON, R. G., M.B. Lond. | 119 Redland Road, Bristol 6. |
| *JOLL, C. A., M.D., M.S. Lond. | 64 Harley Street, London, W.1. |
| *JORDAN, L. R., M.B., Ch.B. Brist. | National Temperance Hospital, Hampstead Road, N.W.1. |
| *JOSCELYNE, P. C., M.B., Ch.B. Brist. | 70 Longmead Avenue, Bishopston, Bristol 7. |
| *KEMM, N. F., M.B., B.S. Lond. | 200 Redland Road, Durdham Park Bristol 6. |
| KING, E. F. | 2 Upper Wimpole Street, London, W.1. |
| *KINGSTON, S. H., M.B., Ch.B. Brist. | 3 Whiteladies Road, Clifton, Bristol 8. |
| *KYLE, H. G., M.A., M.D., B.Ch. Oxon. | 31 Westbury Road, Bristol. |
| *LACE, F | 5 Gay Street, Bath. |
| LALONDE, T. P., M.B., Ch.B. Brist. | Wykeham House, Romsey, Hants. |
| *LEES, E. LEONARD, M.D., C.M. Edin. | 22 Richmond Hill, Clifton, Bristol 8. |
| *LEGAT, R. EDDOWES, M.B., C.M. Edin. | Murray House, Staple Hill, Bristol. |
| *LEWIS, F. J., M.B., Ch.B. Brist. | The Royal Infirmary, Bristol. |
| *LING, T. M., M.A., B.M., B.Ch. | "St. Budoc," Elmlea Avenue, Stoke Bishop, Bristol. |
| *LINTON, MARION S., M.B. Lond. | 1 Brecon Road, Henleaze, Bristol. |
| *LISTER, A. E. J., M.B., B.S. Lond. | 86 Pembroke Road, Clifton, Bristol 8. |
| *LISTER, L. MARGARET | 12 All Saints Road, Clifton, Bristol 8. |
| *LOGAN, H. B. | Elmwood, Aberdeen Road, Cotham, Bristol 6. |
| *LOXTON, S. D., M.B., Ch.B. | 17 Oakfield Grove, Clifton, Bristol 8. |
| *LUCAS, J. E. | 48 Coronation Road, Bristol 3. |
| *LUCAS, J. J. S., M.D. Lond. | 186 Wells Road, Totterdown, Bristol 4. |
| *LUCAS, J. V. | 186 Wells Road, Knowle, Bristol 4. |
| *LUCAS, R. P., M.B., Ch.B. Brist. | 15 Edgecumbe Road, Redland, Bristol 6. |
| *MCCREA, PHILLIP, M.D., B.Ch. Dub. | 56 Kellaway Avenue, Bristol 6. |
| *MCKENZIE, W. G., *M.C.* | 10 Woodland Road, Surbiton. |
| *MACLACHLAN, A. M., M.B., Ch.B. Glasg. | 65 Redcliff Hill, Bristol 1. |
| *MCLANNAHAN, J. G. | The Mount, Stonehouse. |
| MACLEAN, SIR EWEN J., M.D., C.M. Edin. | 12 Park Place, Cardiff. |
| *MACLEOD, DONALD, M.B., C.M. Edin. | 84 Redland Road, Redland, Bristol 6. |
| *MACLEOD, G., M.A. Glas., M.D., M.B. | Wargrave, Clevedon, Somerset. |
| *MACWATTERS, J. C., *M.B.E.* | Almondsbury, near Bristol. |
| *MARSHALL, A. H., M.B., Ch.B. Brist. | Odelos, Canford Lane, Bristol. |
| MARSHALL, A. L., M.B., B.A. Cantab. | Laxfield, Woodbridge, Suffolk. |
| *MARTIN, J. J. B., M.A., M.D. Edin. | Bristol Mental Hospital. |
| *MARTIN, P. S., *M.C.* | Victoria Quadrant, Weston-super-Mare. |
| *MAYES, F. J A. | 23 Pembroke Road, Clifton, Bristol 8. |

| | |
|---|---|
| *Meadows, G. .. .. .. .. .. .. .. .. .. | W. D. & H. O. Wills, No. 1 Factory, East Street, Bedminster, Bristol 3. |
| Miall, C. L'O .. .. .. .. .. .. .. .. .. | Elm Hayes, Paulton, near Bristol. |
| *Milling, T., M.B., Ch.B. Belf. .. .. .. .. | 1 Vicarage Road, Southville, Bristol 3. |
| *Milner, A., M.B., B.Ch. Dub. .. .. .. .. | 61 Cotham Brow, Bristol 6. |
| *Moore, C. A., M.B., M.S. Lond. .. .. .. | 56 St. Paul's Road, Clifton, Bristol 8. |
| *Moore, L. A., M.B., Ch.B. Brist. .. .. .. | Hillsborough, Cotham Park, Bristol 6. |
| *Moore, R. H., M.B., Ch.B. Brist. .. .. .. | White Cross Court, Wells Road, Bristol 4. |
| *Morgans, C. C., M.B., Ch.B. Cantab. .. .. | 65 Lower Redland Road, Bristol 6. |
| *Morris, L. N., M.B., Ch.B. Brist. .. .. .. | 13 Belgrave Road, Clifton, Bristol 8. |
| *Mundy, G. S., M.B., B.Ch. Brist. .. .. .. | Homewood, Coombe Dingle, Bristol. |
| *Myles, G. T... .. .. .. .. .. .. .. .. .. | 7 Apsley Road, Clifton, Bristol 8. |
| *Myles, Q. St. L., M.A., B.M., B.Ch. Oxon. .. | 7 Apsley Road, Clifton, Bristol 8. |
| *Newlands, H. J. .. .. .. .. .. .. .. | Moray House, Fishponds, Bristol. |
| *Nicholson-Lailey, J. R. .. .. .. .. .. .. | 8 Mount Terrace, Taunton. |
| *Nixon, Doreen, M.B., B.S. Lond. .. .. .. | 7 Lansdown Place, Clifton, Bristol 8. |
| *Nixon, J. A., *C.M.G.*, M.D. Cantab. .. .. | 7 Lansdown Place, Clifton, Bristol 8. |
| *Noble, J. I., M.B., Ch.B. Liverp. .. .. .. | 102 Pembroke Road, Clifton, Bristol 8. |
| *Norman, R. M., M.D. Brist. .. .. .. .. .. | 25 Victoria Square, Clifton, Bristol 8. |
| *Nott, Winifred, M.B., Ch.B. Brist. .. .. | Fassiefern, 8 Rylestone Grove, Stoke Bishop, Bristol 9. |
| *O'Donohoe, Monica A., M.B., Ch.B. .. .. | 19 Westfield Park, Redland, Bristol 6. |
| *Oliver, L. C. .. .. .. .. .. .. .. .. | The General Hospital, Bristol. |
| *Ormerod, G. L. M.A., M.B., B.Ch. Cantab .. | 2 Henleaze Road, Westbury-on-Trym, Bristol. |
| *Orr-Ewing, H. J., *M.C.*, M.D. Lond. .. .. | 1 Beaufort Buildings, Clifton, Bristol 8. |
| *Parkes, J. A., M.B., Ch.B. Liverp. .. .. | 8 South Road, Redland, Bristol 6. |
| Parkinson, Lt.-Col. G. S., *D.S.O.*, R.A.M.C. | R.A.M.C. Mess, Grosvenor Place, London, S.W.1. |
| *Parry, R. H., M.B., B.S. Lond. .. .. .. | Public Health Offices, 40 Prince Street, Bristol 1. |
| Parsons, Sir J. H., M.B., B.S., B.Sc. Lond... | 54 Queen Anne Street, London, W. |
| *Paul, R. G. .. .. .. .. .. .. .. .. .. .. | 23 Pembroke Road, Bristol 8. |
| *Perry, B., M.D., Ch.B. Brist. .. .. .. .. | 4 Richmond Park Road, Clifton, Bristo 8. |
| *Philipp, E., M.B., Ch.B. .. .. .. .. .. | General Hospital, Bristol. |
| *Phillips, L. B., M.B., Ch.B. Brist... .. .. | Holdenhurst, Filton Bristol. |
| *Phillips, P., M.B., Ch.B. Brist. .. .. .. | Southmead Hospital, Bristol. |
| *Pineo, E. D. .. .. .. .. .. .. .. .. | Richmond House, Langford, Som. |
| *Pollard, J., M.B., B.Ch., B.A.O., N.U.I .. | 88 Coronation Road, Bristol 3. |
| Porter, C. P. .. .. .. .. .. .. .. .. .. | 28 Mill Street, Kidderminster. |
| *Potter, Mabel F., M.B., Ch.B. Brist. .. .. | 23 Pembroke Road, Clifton, Bristol 8. |
| *Powell, H. F., M.D. Brux. .. .. .. .. .. | Ellenborough Park, Weston-super-Mare. |
| *Price, C. H. G., M.B., Ch.B. Brist. .. .. | 6 Lansdown Place, Bristol 8. |
| *Price, N. L., M.B., Ch.B. Brist. .. .. .. | Southmead Hospital, Bristol. |
| *Pridie, K. H., M.B., B.S. Lond. .. .. .. | 58 St. John's Road, Clifton, Bristol 8. |
| *Pringle, Miss J. L., M.B., Ch.B. Edin. .. | 115 Lower Oldfield Park, Bath. |
| *Prowse, D. C., M.B., Ch.B. Brist. .. .. .. | Wigmore House, Thornbury, near Bristol. |
| *Pyke, H. D., M.B., Ch.B. Brist. .. .. .. | 88 Redland Road, Bristol 6. |
| *Rankin, P. C., M.B., Ch.B. Glasg. .. .. .. | Lawrence Hill House, Bristol. |
| *Rayner, D. C., Ch.M. Brist. .. .. .. .. | 9 Lansdown Place, Clifton, Bristol 8. |
| *Redman, Ethel, M.B., Ch.B. .. .. .. .. | 55 Bridgwater Road, Bedminster Down, Bristol 3. |
| *Roberts, J. A. F., M.A. Cantab., M.B., Ch.B., D.Sc. Edin. | Stoke Park Colony, Bristol. |
| *Robertson, D., M.B., Ch.B. Edin. .. .. .. | 2 Knowle Road, Knowle, Bristol 4. |
| *Robertson, L. G., M.B., Ch.B. .. .. .. | 162 Redland Road, Bristol 6. |
| *Rogers, H., M.D. Brist. .. .. .. .. .. | 91 Old Park Ridings, Grange Park, N.21. |
| *Rolfe, R., B.A., M.B., B.C Cantab. .. .. | Park House, St. Andrew's Road, Avonmouth. |
| *Rowley, A. T. .. .. .. .. .. .. .. .. | 13 Walliscote Road, Weston-super-Mare. |
| *Rudolf, G. R. A. de M. .. .. .. .. .. | West Hythe, Patchway, Glos. |
| *Scarff, G., M.B., Ch.B. Edin. .. .. .. .. | 83 Pembroke Road, Clifton, Bristol 8. |
| *Shaw, J. E., M.B., C.M. Edin. .. .. .. | 23 Caledonia Place, Clifton, Bristol 8. |

| | |
|---|---|
| *SHEPHERD, H. L., M.B., Ch.M. Brist. .. .. | 79 Pembroke Road, Clifton, Bristol 8. |
| *SHORT, A. R., B.Sc., M.D., B.S. Lond. .. .. | 69 Pembroke Road, Clifton, Bristol 8. |
| *SMITH, T. W., M.D. Lond... .. .. .. .. .. | 26 The Circus, Bath. |
| *SMYTHE, H. J. D., *M.C.*, M.D., M.S. Lond. .. | 15 Eaton Crescent, Clifton, Bristol 8. |
| *SOLTAU, H. K. V., M.D. Lond. .. .. .. | 19 The Avenue, Clifton, Bristol 8. |
| *SOMERS, MARY A. E. .. .. .. .. .. .. .. | 2 Ashcombe Road, Weston-super-Mare. |
| *SPOOR, A., M.B., B.Ch. Cantab. .. .. .. | The Hydro, Bristol 1. |
| *STANILAND, M. F... .. .. .. .. .. .. .. | 255 Stapleton Road, Bristol 5. |
| *STATHAM, R. S. S., *O.B.E.* M.D., M.Ch. Brist. | Cheddar, Som. |
| *STEVEN, G. D., M.B., Ch.B. Edin., D.P.H. .. | 6 Upper Church Street, Bath. |
| *STONE, D. M., M.D., B.S. Lond. .. .. .. | Manor House, York Place, Clifton, Bristol 8. |
| *STROVER, H. W. M., *O.B. E.*, M.B., Ch.B. Aberd. | 12 Gloucester Road, Bishopston, Bristol 7. |
| *STRUTHERS, A. J., M.B., Ch.B. Glasg. .. .. | 100 Church Road, Redfield, Bristol 5. |
| *SUTTON, G. E. F., M.D. Lond. .. .. .. .. | 1 Pembroke Road, Clifton, Bristol 8. |
| *SYMES, J. O., M.D. Lond. .. .. .. .. .. | 6 Pembroke Vale, Clifton, Bristol 8. |
| *TASKER, D. G. C., M.B., M.S. Lond. .. .. | 9 College Fields, Clifton, Bristol 8. |
| *TAYLOR, A. L., M.D. Lond. .. .. .. .. | The General Hospital, Bristol 1. |
| TAYLOR, A. L. .. .. .. .. .. .. .. .. | The Royal Infirmary, Bristol 1. |
| *TAYLOR, CONSTANCE L., M.B., Ch.B. Edin. .. | Queen Charlotte's Hospital, Marylebone Road, N.W.1. |
| *TAYLOR, H. N., M.A. Cantab., M.D. Dub. .. | Hillside, Ebbw Vale. |
| *TEMPLE, G. H., M.B., C.M. Edin. .. .. .. | Ailanthus, Weston-super-Mare. |
| THIN, J... .. .. .. .. .. .. .. .. .. | 54 and 55 South Bridge, Edinburgh. |
| *THOMAS J. D., M.B. Cantab. .. .. .. .. | Dan-y-Graig, Cheyne Road, Stoke Bishop, Bristol 9. |
| *TODD, A, T., *O.B.E.*, M.D. Edin. .. .. .. | Royal Park House, Clifton, Bristol 8. |
| TRIPP, DOROTHY M. H. .. .. .. .. .. .. | Winthill, Banwell, Somerset. |
| *TRIST, J. R. R., *M.C.* .. .. .. .. .. .. | 120 Henleaze Road, Henleaze. |
| *TRYON, VICTORIA S., M.B., Ch.B. Brist. .. | 3 Harley Place, Clifton, Bristol 8. |
| *WALKER, CYRIL H., B.A., M.B. Cantab. .. | Harewood, Clapton-in-Gordano, Som. |
| *WALTERS, C. F. .. .. .. .. .. .. .. .. | Downover, Walton Down, near Clevedon. |
| *WANSBROUGH, T. B., M.B., Ch.B. Brist. .. | 8 Mortimer Road, Clifton, Bristol 8. |
| WAKE, S. C., M.B., Ch.B. Brist. .. .. .. | Brantwood, Bideford, N. Devon. |
| *WARD, H. W. .. .. .. .. .. .. .. .. .. | Chipping Manor, Wootton-under-Edge. |
| *WARREN, R., M.A., M.D. Oxon. .. .. .. | Heathgates, Weston-super-Mare. |
| *WATERHOUSE, R,, M.D. Lond... .. .. .. .. | 3 The Circus, Bath. |
| *WATSON-WILLIAMS, E., *M.C.*, B.A., M.B., Ch.M. Cantab. | 65 Pembroke Road, Clifton, Bristol 8. |
| WATTS, J. B. V. .. .. .. .. .. .. .. .. | 6 Queen Street, Cambridge, C.P., South Africa. |
| WEDDELL, C. W. .. .. .. .. .. .. .. .. | Boydville, 46 Miller Street, West Melbourne, Australia. |
| WHITEHOUSE, H. B., M.S. Lond. .. .. .. | 62 Hagley Road, Edgbaston, Birmingham. |
| *WIGODER, SYLVIA BEATRICE, M.A., M.D., Ph.D. | Radium Centre, Royal Infirmary, Bristol 1. |
| *WILBOND, R. G. .. .. .. .. .. .. .. .. | 92 Chesterfield Road, St. Andrew's, Bristol. |
| *WILLIAMS, I., M.B., Ch.B. Brist. .. .. .. | "Ardrem," Hill View, Henleaze, Bristol. |
| WILLIAMS, S. R., M.B. Lond. .. .. .. .. | Buckfastleigh, Devon. |
| *WILLS, W. K., *O.B.E.*, M.B., B.C. Cantab. .. | The Old Vicarage, Henbury, Bristol. |
| *WILLWAY, F. W., M.D., M.S. Lond. .. .. | Bristol Royal Infirmary. |
| *WOOD, D. .. .. .. .. .. .. .. .. .. .. | 105 Pembroke Road, Clifton, Bristol 8. |
| *WOOD, W. V., *M.C.*, B.A. Cantab. .. .. .. | Henley Lodge, Yatton, Som. |
| *WOODMAN, DOROTHY, M.D. Lond. .. .. .. | Kynance, Downs Cote Avenue, Westbury-on-Trym, Bristol. |
| *WOOLLEY, A. W., M.B., Ch.B. Brist. .. .. | Melbourne House, Wells, Som. |
| *WOOLLEY, W., M.B., Ch.B. Brist. .. .. .. | 239 Cranbrook Road, Redland. |
| *WORTHINGTON, Lieut.-Col. F., M.B., Ch.B. Cantab. | Greytrees, Briercliffe Road, Coombe Dingle, Bristol 9. |
| *WRIGHT, A. J., M.B., B.S. Lond. .. .. .. | 2 Clifton Park, Clifton, Bristol 8. |
| *WRIGHT, WINIFRED M., M.B., B.S. Lond. .. | Merrymead, Shepton Mallet, Som. |
| *ZEALAND, L., M.D. Man. .. .. .. .. .. .. | Brecon Lodge, Westbury-on-Trym, Bristol. |

